# Diagnostic accuracy of a self-administered digital cognitive test battery for differentiating early Alzheimer’s disease from controls

**DOI:** 10.1007/s00406-026-02347-2

**Published:** 2026-08-17

**Authors:** Sophia Rutt, Paulina Tegethoff, Marleen Taute, Fortese Neziraj, Anna Hufnagel, Robert Perneczky, Carolin Kurz, Boris-Stephan Rauchmann

**Affiliations:** 1 Department of Psychiatry and Psychotherapy, LMU University Hospital, LMU Medizin, Ludwig-Maximilians-Universität München, Nußbaumstr. 7, 81377 Munich, Germany; 2 German Center for Neurodegenerative Diseases (DZNE), 81377 Munich, Germany; 3 Cluster for Systems Neurology (SyNergy), 80336 Munich, Germany; 4 Department of Neuroradiology, LMU University Hospital, LMU Medizin, Ludwig-Maximilians-Universität München, 81377 Munich, Germany; 5 Ageing Epidemiology (AGE) Research Unit, School of Public Health, Imperial College London, London, W6 8RP UK; 6 Sheffield Institute for Translational Neuroscience (SITraN), University of Sheffield, Sheffield, S10 2HQ UK; 7 Department of Psychiatry and Psychotherapy, School of Medicine and Health, Technical University Munich , Ismaninger Str. 22, 81675 Munich, Germany

**Keywords:** Digital cognitive assessment, Early Alzheimer's disease, Self-administered testing, Early Detection, Cognitive Impairment

## Abstract

**Background:**

Paper-pencil assessments face limitations in accessibility, lengthy administration, and interrater reliability. Digital tools may overcome these by offering user-friendly, non-verbal, and scalable assessments. This study investigated the diagnostic accuracy of BraincheX, a novel digital test battery focused on accessibility and usability, by comparing its subtest performance to CERAD-Plus equivalents.

**Methods:**

Fifty-six participants with early AD and amyloid negative cognitively healthy controls were recruited from the LMU hospital memory clinic. Diagnostic groups were defined by amyloid positivity and clinical expert consensus according to the IWG-2 criteria. Participants completed the BraincheX digital battery and CERAD-Plus. BraincheX total score was calculated by summing standardized subtest scores. Data were analyzed using ROC analysis and the Youden Index. Correlations between subtests were examined, and group differences analyzed via ANOVA.

**Results:**

ROC analysis revealed strong diagnostic accuracy for the BraincheX total score in differentiating between early AD and controls (AUC = 0.86; optimal cut-off = − 1.51), with high sensitivity of 89.7% and moderate specificity of 68.0%. BraincheX total scores correlated with CERAD total scores (*r* = .73, *p* < .001). BraincheX demonstrated good internal consistency (Cronbach’s α = 0.84). ANOVA confirmed significant group differences across all BraincheX subtests (*p* < .01). Individual subtests showed strong correlations with CERAD-Plus equivalents.

**Conclusions:**

Findings suggest BraincheX as a promising, accessible digital screening tool for cognitive decline, offering automated scoring, immediate feedback, and reduced examiner bias. Future research is required to validate BraincheX in larger and more diverse populations and to establish BraincheX’s clinical applicability.

**Trial Registry:**

This analysis was conducted within the framework of the prospectively registered German SCD-Q validation study (NCT06711952). The registered study protocol included digital cognitive testing as part of the assessment protocol. The present manuscript reports a secondary analysis focusing on the diagnostic performance of BraincheX.

**Supplementary Information:**

The online version contains supplementary material available at https://doi.org/10.1007/s00406-026-02347-2.

## Introduction

Effective clinical management of Alzheimer´s Disease (AD) begins with an early and specific assessment, as neuropathological changes often precede symptoms by years [1]. Early diagnosis empowers individuals to actively manage their health, make informed decisions, and engage in personalized preventive strategies, aiming to slow disease progression [1]. It also opens opportunities to maintain autonomy, build resilience, and preserve quality of life for as long as possible [2].

Standardized cognitive assessments, such as the Consortium to Establish a Registry for Alzheimer’s Disease (CERAD)-Plus test battery, the Montreal Cognitive Assessment (MoCA), and the Mini-Mental-State Examination (MMSE) are well established in both clinical and research settings. However, these assessments were developed for rater-administered cognitive screenings, limiting their more widespread use. Such limitations include the absence of remote administration capabilities, restricted potential for longitudinal assessment, lack of integration of multimodal data, and reliance on trained raters, which introduces potential for rater bias and variability in administration and scoring [3]. Most traditional paper-and-pencil cognitive instruments tend to be more sensitive to deficits in later disease stages and are typically administered in specialized clinical settings, which can limit their utility for early detection and broad accessibility. More recent tools like the MoCA offer improved sensitivity for earlier stages but still rely on trained personnel and structured environments [3, 4].

Digital cognitive test batteries offer a promising alternative to paper-and-pencil methods by enabling precise measurement of response times, standardized stimulus presentation, and automated comparison to individual baselines and normative data [3, 5, 6]. Depending on their implementation, they may improve efficiency, support self-administration, and require less examiner involvement through standardized administration and scoring procedures [3]. In addition, digital tools have the potential to capture nuanced behavioral data, such as spatial planning strategies, and provide immediate, analyzable feedback for clinicians and participants [3, 6, 7].

In this study we assessed the performance in differentiating early AD individuals from amyloid negative cognitively healthy controls of BraincheX (Medotrax GmbH, Munich, Germany), a novel digital test battery designed to evaluate cognitive function across multiple domains, including complex attention, executive function, learning and memory and perceptual-motor function. These cognitive domains incorporate factors such as cognitive flexibility, processing speed, visual memory, and planning skills. BraincheX offers a non-verbal format that minimizes the impact of language barriers and integrates digital variants of traditional tests alongside a newly developed visual memory test. Through standardized administration and partly automated scoring, BraincheX is designed to minimize examiner bias and captures data relevant to everyday functioning. Through a direct comparison of BraincheX subtests and their CERAD-Plus equivalents, this study assesses the diagnostic performance of BraincheX in identifying cognitive impairment. First the comparison of these two assessments helps to evaluate the overall outcome of the BraincheX test battery, as well as look at the subtests with heir corresponding CERAD-Plus measures to examine their diagnostic validity.

BraincheX intends to offer a digital, self-administered alternative that enables individuals to complete cognitive testing remotely, without requiring a clinic or general practice visit. By minimizing language dependency, BraincheX is intended to facilitate cognitive assessment across individuals with diverse linguistic and cultural backgrounds, thus improving diagnostic accessibility and inclusivity in the general population. Moreover, unlike many existing tools that target only singular cognitive domains, BraincheX encompasses a broad range of domains within one unified test battery. Its standardized, partly automated scoring and user-friendly interface are intended to support reproducibility, scalability, and applicability in both research and clinical settings. In this current study BraincheX was evaluated under supervised administration.

## Methods

### Participants

Participants were recruited consecutively from the memory clinic at the Department of Psychiatry and Psychotherapy, LMU University Hospital, LMU Medizin, Ludwig-Maximilians-University Munich, between October 2024 and February 2025. Participants entered the study after visiting the Alzheimer Therapy and Research Center for diagnostics, due to memory complaints or suspected cognitive impairment. All individuals presented for a standard diagnostic work-up due to memory complaints or suspected cognitive impairment. A total of 56 participants meeting the inclusion criteria were enrolled and completed both the BraincheX and CERAD-Plus test batteries. Inclusion criteria encompass the following: (1) referral for cognitive evaluation, (2) provision of signed, written, and dated informed consent, (3) confirmed capacity to consent, (4) age of 50 years or older, and (5) expert diagnosis of early AD according to the German S3 Dementia Guideline (2023) — classified as mild cognitive impairment (MCI) or mild dementia due to AD — or as an age-matched cognitively healthy control [1]. MCI was defined by objective cognitive deficits without significant impairment of daily functioning. Early AD diagnosis adhered to the IWG-2 criteria, requiring both clinical phenotype and biomarker evidence (amyloid positivity via cerebrospinal fluid [CSF] analysis and/or positron emission tomography [PET]). Exclusion criteria included (1) fewer than nine years of formal education, (2) diagnosis of any neurodegenerative disease other than AD, and (3) moderate to severe dementia stages. Diagnostic classification was determined by clinical expert consensus and supported by biomarker profiles [1]. For the following analysis, participants were categorized into two groups: amyloid-positive early AD (*n* = 25; mild AD dementia *n* = 12; MCI-AD *n* = 13) and amyloid-negative cognitively healthy controls (*n* = 31). The cognitively healthy control group consisted of individuals who underwent the same diagnostic work-up at the memory clinic but did not meet the diagnostic criteria for MCI or dementia and showed no biomarker evidence of AD.

Participants were contacted via telephone and recruited through convenience sampling. Travel expenses were reimbursed. All participants were fully informed about the study procedures and provided written informed consent. The capacity to consent was ensured by confirming adequate cognitive function based on investigator judgment during the consent process. The study was approved by the Ethics Committee of LMU Munich (project number 22–1117) and is registered at ClinicalTrials.gov (NCT06711952).

## Evaluation of the digital neurocognitive test battery BraincheX

BraincheX is a mobile application for cognitive assessment, developed by Medotrax GmbH, Munich, Germany, offering digital variations of established paper-and-pencil tests alongside a newly developed visual memory task. It is designed for efficient, accessible cognitive screening, with an average completion time of 23.60 minutes depending on the participant’s cognitive state.

The BraincheX battery includes (additional details are provided in Supplementary Table S1):

- A modified Symbol Digit Test (SZT), similar to the RBANS and WAIS-IV subtests, presenting one practice example followed by assignments across 9 symbols within a 60-second time limit. This subtest assesses processing speed and attention. Participants are presented with a key containing nine digit–symbol pairs and are instructed to match each digit shown on screen with its corresponding symbol as quickly and accurately as possible within a 60-second time limit. One practice trial ensures understanding of the procedure. During the test phase, a single digit appears on the screen, and participants select the corresponding symbol from multiple options by tapping the correct one on the touchscreen. After each response, the next digit is presented automatically. The task continues sequentially until the time limit is reached. The final score represents the number of correct responses. The task structure and scoring are comparable to the *Digit–Symbol Coding* subtest of the WAIS-IV and the *Coding* subtest of the RBANS, with age-, education- and sex-adjusted normative data adapted from these instruments. Performance is scored as the number of correct responses, with adapted normative references based on age groups [8, 9].
- A Visual Memory Test (VMT), presenting 20 images for encoding and subsequently 35 images (mix of new and familiar), requiring recognition decisions. The task structure is unique, though comparable to aspects of the Wechsler Memory Scale (WMS-IV Visual Reproduction) and the Visual Paired Comparison (VPC) tasks [10]. This task measures visual episodic memory and recognition accuracy. In the encoding phase, participants view 20 color images (everyday objects and organisms) presented in a slideshow format. In the recognition phase, 35 images (20 previously shown and 15 novel images) are displayed sequentially in randomized order. For each image, the participant must indicate whether it was seen before (“Yes”) or is new (“No”) by tapping corresponding buttons. Accuracy and response times are recorded. Normative data were acquired within the current project, as no directly equivalent test existed previously. The structure of the VMT and additional memory recognition tasks mimic the word recognition paradigms used in the CERAD, incorporating principles of discriminability without the language dependence [10–14].
- A Line Orientation Test similar to the RBANS structure, where 10 pairs of lines must be matched for orientation within 15 seconds per item. Scores are compared against adjusted norms to account for the added time constraint [8, 9]. This subtest evaluates visuospatial perception. Two target lines are displayed at the top of the screen, and the participant must select the matching pair from 10 lines shown below, which differ in angular orientation. Responses are made by choosing the number corresponding to the correct line pair within 15 seconds per item. Ten trials are administered. Performance is scored as the number of correctly identified pairs [8, 9].
- A digital Pathfinding Test comparable with the Trail Making Test Part B (TMT-B), in which participants are required to alternately connect 13 numbers and letters in ascending order. Participants are presented with 13 numbered circles (“1” to “13”) and 13 lettered circles (“A” to “M”) positioned randomly on the screen. They must alternately connect numbers and letters in ascending order (“1–A–2–B–3–C … M”) by tapping each target in sequence. The program automatically records time to completion, while errors delay completion, with a maximum duration of 300 seconds. Trials exceeding this limit are automatically capped [15, 16].
- A digital version of the Clock Drawing Test rated by using the Shulman’s procedure, requiring participants to depict “10 past 11” on a pre-drawn circle following detailed standardized instructions [13]. This subtest assesses visuoconstructional ability and executive planning. Participants receive on-screen written and verbal instructions to “Draw a clock that shows the time 10 past 11.” The display shows a pre-drawn circle representing the clock face. Participants draw the numbers and clock hands directly on the touchscreen using an Apple Pencil or finger. In this study the drawing is automatically stored and later rated according to Shulman’s scoring procedure, assessing contour completeness, number placement, and accuracy of time depiction [13]. An automated algorithm for scoring the Clock Drawing Test has been developed but was not applied in the present study.

As an established neuropsychological assessment, the CERAD-Plus battery was used, comprising tests for verbal fluency, naming, memory encoding and recall, recognition memory, visuoconstruction, and executive function (Table 1, additional details are provided in Supplementary Table S2). Subtests include Animal Naming, Phonemic Fluency, Trail Making Test A and B, Word List Learning, Recall and Recognition, Figure Recall, Modified Boston Naming Test, and Figure Copy. CERAD-Plus requires approximately 30 to 40 min to administer and is widely applied in clinical and research contexts for detecting and tracking cognitive decline [15, 16].

**Table 1:** Comparison CERAD-Plus vs. BraincheX

Feature	CERAD-Plus	BraincheX
	Paper-pencil	Fully digital
Domains assessed	Memory, language, visuoconstruction, executive functions	Complex attention, executive function, learning, memory, perceptual-motor function
Test-taking Time	30 to 40 min	15 to 25 min
Administration	Examiner-administered	Possibility of self-administration with minimal supervision
Scoring	Manual scoring, prone to interrater variability	Immediate results calculation
Language dependence	Language-heavy	Language-independent (e.g., visual memory test)
Setting	Specialized memory clinics	Scalable and broader, decentralized deployment

This table compares the features of the CERAD-Plus and BraincheX test battery regarding Domains assessed, Test-taking time, Administration, Scoring, Language dependence, Ecological validity and Setting

## Study design

The aim of this prospective, non-interventional, cross-sectional pilot study was to compare the performance and usability of the BraincheX and CERAD-Plus test batteries. Participants meeting the inclusion criteria were recruited to complete the BraincheX and CERAD-Plus test battery. For comparison, CERAD-Plus paper-based assessments conducted in the same patients within an average interval of 4.9 months before the BraincheX testing [7]. Participants completed the CERAD-Plus as part of the standard diagnostic procedures at the LMU hospital memory clinic. Within an average of 4.9 months (range from 0 to 12 months), participants then completed the BraincheX test battery.

The BraincheX test battery was administered in a controlled setting, with a trained psychologist present to assist if necessary and to ensure standardization across participants. Participants completed the BraincheX test battery in a quiet environment. An Apple iPad (iOS 17.5.1) and Apple Pencil Pro were provided to ensure standardized input conditions. Participants were instructed to carefully read the on-screen instructions and could ask the examiner if questions arose. Sociodemographic information such as age, sex, years of education and occupation were obtained. Test results were automatically recorded and processed by the BraincheX application (except Clock Test results). Data from the CERAD-Plus test battery were obtained from clinical records or prior research participation. These assessments had been administered by trained professionals in clinical or research settings according to standard protocols.

### Statistical analysis

Pseudonymized data from BraincheX and paper-based questionnaires were analyzed using IBM SPSS Statistics [17]. Due to missing data, two participants were excluded (final *N* = 54) because incomplete BraincheX results prevented calculation of the total score. Missingness was not considered to be systematically related to diagnostic group. Descriptive characteristics (education, age, marital status, diagnosis) were calculated for initial group comparisons between AD and controls. Following the principles of CERAD-Plus total score calculation, BraincheX also calculates a total score (BraincheX score). For CERAD-Plus, six core subtests (Animal Naming, Boston Naming, Word List Learning, Recall, Recognition, Figure Copy) were included, excluding domains primarily relevant for Parkinson’s disease differentiation (e.g., TMT-A/B, phonemic fluency, figure recall) [18–20]. The BraincheX score was calculated as the sum of the z-scores of all subtests. Prior to summation, scores were aligned so that higher values consistently reflect better cognitive performance. The Clock Drawing Test and TMT-B completion time were inverted, as higher values on these measures indicate poorer performance.

Z-scores were computed based on control group normative data (*N* = 29; additional details are provided in Supplementary Table S3). For each BraincheX subtest, age-, sex-, and education-adjusted scores were derived using regression models fitted to the amyloid negative cognitively healthy control group. Standardized residuals from these models were used as demographically corrected z-scores, which were summed across the five subtests (SZT, VMT, LOT, TMT-B, Clock Drawing Test) ^13^. Z-scores were calculated to account for the different measurement scales of the subtests. Internal consistency was evaluated using Cronbach’s α, calculated based on the standardized (z-transformed) subtest scores. Standardization placed all subtests on a common metric, thereby minimizing the influence of differences in measurement scales. The resulting Cronbach’s alpha reflects the consistency of the subtests contributing to the total BraincheX score and should be interpreted accordingly. Diagnostic accuracy was assessed via receiver operator (ROC) curve analyses for each subtest and the BraincheX score. Study specific cut-offs were determined using the Youden Index. Correlations between corresponding BraincheX and CERAD-Plus subscores were analyzed, and differences between diagnostic groups were tested with univariate ANOVA adjusted for age, sex and education and post-hoc comparisons. The control, MCI and AD group were separated before using univariate ANOVA, resulting in comparisons across three diagnostic groups. To assess convergent validity, partial correlations between BraincheX and CERAD-Plus subtests were calculated while controlling for age, sex, and education. The subtest analyses were exploratory; therefore, no correction for multiple comparisons was applied.

## Results

### Demographic characteristics

The study cohort included 21.4% of participants diagnosed with AD dementia (*n* = 12), 23.2% with MCI (*n* = 13), and 55.3% (*n* = 31) were amyloid negative cognitively healthy controls. For statistical analysis two participants were removed from the amyloid negative cognitively healthy controls (*n* = 29). This distribution reflects a balanced representation of individuals across the continuum of cognitive functioning, providing a suitable sample for evaluating the diagnostic accuracy of BraincheX compared to traditional assessments. The final sample consisted of 56 participants aged between 50 and 87 years. The majority of the cohort was female (66.1%) and most participants (69.6%) were retired, 14.3% were actively employed, and smaller proportions were homemakers (5.4%), disability pensioners (5.4%), or self-occupied/other (5.4%) (Table 2).

**Table 2:** Descriptive Statistics of the enrolled Study Population (*N* = 56)

Variable	Early AD (*n* = 25)	Control (*n* = 31)	*p*-value
Age (range, years)	50–86	50–87	0.19
Education			0.27
Primary	8 (32%)	3 (9.7%)	
Secondary	8 (32%)	16 (51.6%)	
Tertiary	9 (36%)	12 (38.7%)	
Sex			0.39
Female	15 (60%)	22 (71%)	
Male	10 (40%)	9 (29%)	
Marital Status			0.05
Married	16 (64%)	15 (48.4%)	
Divorced	2 (8%)	10 (32.3%	
Single	1 (1%)	4 (12.9%)	
Widowed	4 (16%)	2 (6.5%)	
Domestic Partnership	2 (8%)	0	
Occupational Status			0.02
Retired	17 (68%)	21 (67.7%)	
Occupied (employee)	1 (4%)	7 (22.6%)	
Unoccupied (homemakers)	3 (12%)	0	
Other	1 (4%)	3 (9.7%)	
Disability pensioner	3 (12%)	0	

This table demonstrates the demographic characteristics of the study population. All p-values are based on two-tailed tests. No significant difference in age between the early AD and control group (*p* =.19). No significant difference in education between the early AD and control group (*p* =.27). No significant difference in sex between the early AD and control group (*p* =.39). A significant difference was found in marital status between early AD and control group (*p* =.05). A significant difference was found in occupation between early AD and control group (*p* =.02)

### Performance of the control group on BraincheX

The average completion time in minutes for the BraincheX test battery was *M* = 23.60, *SD* = 11.50, *range* = 11–68 min. The normative data of BraincheX subtests based on the control group is shown in supplementary Table S3.

#### Group comparisons

**Fig. 1:**
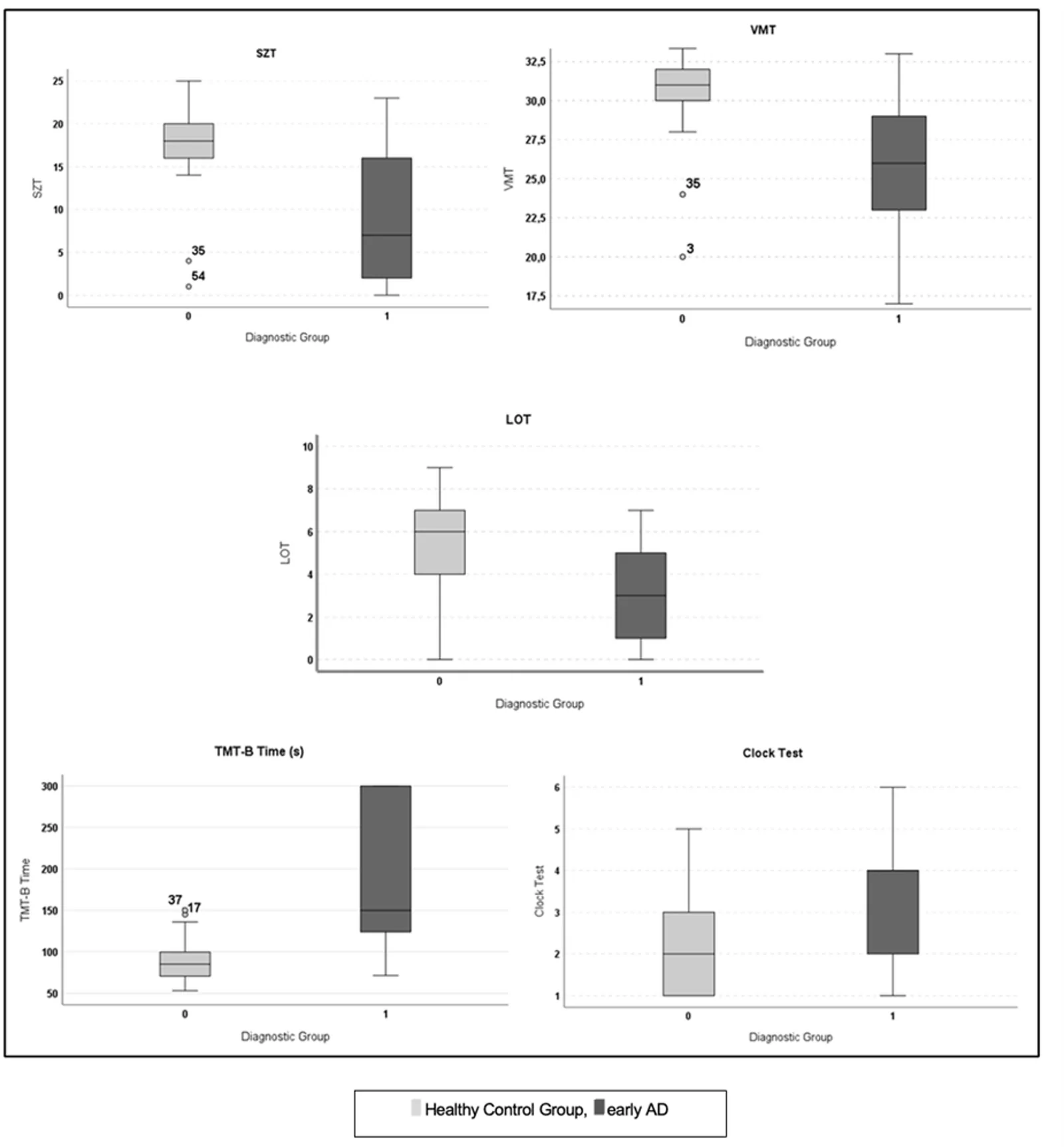
Comparison of Diagnostic Groups based on BraincheX Subtests. Diagnostic group differences in BraincheX Subtests (SZT, VMT, LOT, Clock Drawing Test and TMT-B)

Group differences in BraincheX subtest performance were analyzed using univariate ANOVAs (Fig. 1). Significant differences between diagnostic groups were observed across all subtests: Symbol Digit Test (SZT; *F*(1,54) = 17.60 *p* <.001), Visual Memory Test (VMT; *F*(1,52) = 16.70, *p* <.01), Line Orientation Test (LOT; *F* (1,54) = 11.42, *p* <.01), Trail Making Test B (TMT-B; *F* (1,54) = 30.80, *p* <.001), and Clock Drawing Test (*F* (1,54) = 14.19, *p* <.001). Education consistently showed a significant main effect across all subtests, whereas sex had no significant influence (*p* >.10). Age was only significantly associated with performance on the SZT (*p* <.05). Assumptions of homogeneity of variance were evaluated using Levene’s test. While this assumption was met for most subtests, violations were detected for the VMT and TMT-B (*p* <.05). Accordingly, Welch’s ANOVA was conducted for these subtests, which confirmed significant group effects for the VMT (*F* (1,54) = 12.61, *p* <.001, *η²* = 0.19) and TMT-B (*F* (1,52) = 33.94, *p* <.001, *η²* = 0.40). These results are depicted in Fig. 1.

#### Psychometric properties and diagnostic utility of BraincheX

**Fig. 2:**
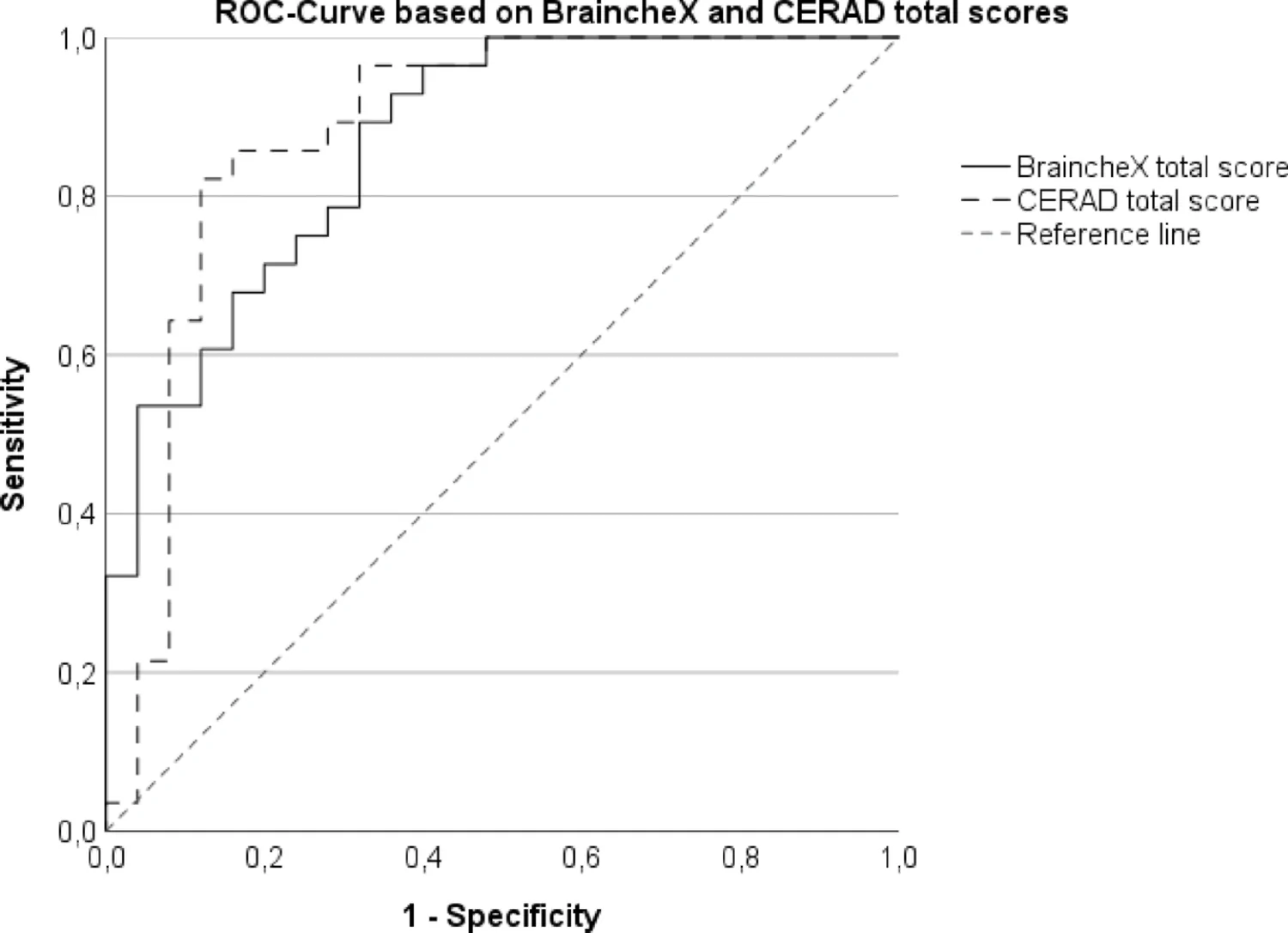
ROC-Curve based on BraincheX and CERAD Total Score. ROC-Curve BraincheX and CERAD total score. Determining the cut-off threshold. Diagonal segments are produced by ties (AUC = .86; 95% CI .76-.95)

To assess the internal consistency of BraincheX, Cronbach’s α was calculated, resulting in an internal consistency coefficient (*Cronbach’s α* = 0.84) [21]. Given the multidomain structure of BraincheX, this coefficient reflects the internal consistency of the total score. Diagnostic accuracy was evaluated through receiver operating characteristic (ROC) curve analysis, resulting in an area under the curve (*AUC*) of 0.86 (95% CI: 0.76–0.95), indicating strong discriminative power between early AD and amyloid negative cognitively healthy controls, comparable to the AUC obtained for the CERAD-Plus (AUC = 88; 95% CI: 0.76–0.97) (additional details are provided in Supplementary Table S4). The optimal cut-off score was determined using the Youden Index, identifying a BraincheX total score threshold of − 1.51. This cut-off yielded a sensitivity of 89.7% and a specificity of 68.0% (Fig. 2). The combination of high sensitivity and moderate specificity suggests that BraincheX may be suitable as a screening instrument for the detection of early cognitive impairment and the identification of individuals who may benefit from further diagnostic evaluation. However, as the cut-off was both derived and evaluated in the same study sample, its diagnostic performance should be interpreted with caution until confirmed in independent cohorts. No internal validation (e.g., bootstrap resampling or cross-validation) was performed. Sensitivity and specificity across a range of candidate cut-off values are reported in Supplementary Table S4.

#### BraincheX and CERAD-plus statistical comparisons

The convergent validity of the BraincheX total score with the CERAD-Plus total score was examined using partial correlations. A strong positive correlation was found (*r* =.73, *p* <.001), suggesting that BraincheX measures domains of cognitive decline closely related to those assessed by established paper-and-pencil batteries.

Associations between BraincheX and corresponding CERAD-Plus subtests were examined using partial correlation analyses, controlling for age, sex, and education. Significant positive correlations were observed between the BraincheX total score and CERAD Clock Drawing Tests (*r* =.47, *p* <.05), TMT-B times (*r* =.44, *p* <.05), and figure construction performance (*r* =.46, *p* <.05). Furthermore, the BraincheX Visual Memory Test (VMT) showed positive associations with several CERAD verbal memory subtests: Word List Recall (*r* =.36, *p* <.05), Animal Naming (*r* =.50, *p* <.01) and Boston Naming (*r* =.49, *p* <.01). These findings are visualized in Table 3, illustrating the strength of associations between specific BraincheX and CERAD-Plus domains of cognitive decline.

**Table 3:** Partial Correlations controlled for age, sex and education

Clock Test CERAD	Clock Test	TMT-B	VMT	SZT	LOT
	0.47 [0.26, 0.68]*	-	-	-	-
TMT-B CERAD	-	0.44 [0.22, 0.66]*	-	-	-
Word List Recall	-	-	0.36 [0.12, 0.59]*	-	-
Word List Learning	-	-	-	0.50 [0.29, 0.70]***	-
Recognition	-	-	-	0.42 [0.19, 0.64]**	0.37 [0.13, 0.60]*
Animal Naming	-	-	0.50 [0.29, 0.70]***	0.62 [0.45, 0.79]***	0.50 [0.29, 0.70]***
Boston Naming	-	-	0.49 [0.28, 0.69]**	-	-
Figure recall	-	-	-	0.42 [0.19, 0.64]**	0.40 [0.17, 0.63]*
Figure drawing	-	-	-	-	0.46 [0.24, 0.67]**

Partial correlations between BraincheX and CERAD-Plus subtests, controlling for age, sex, and education. All confidence intervals represent 95% confidence coefficients

^†^*p* <.10. *p <.05. ** *p* <.01.*** *p*<.001

## Discussion

Reliable and valid digital cognitive assessments are critical to enable early and accurate diagnosis of AD, especially as disease-modifying treatments emerge. In this pilot study, BraincheX demonstrated good psychometric properties, including good discriminative power between early AD and amyloid negative cognitively healthy controls (*AUC* = 0.86), good internal consistency of the total score (*Cronbach’s α* = 0.84), and strong convergent validity with the CERAD-Plus total score (*r* =.73, *p* <.001). These first results support the BraincheX total score as a possible measure of global cognitive functioning. Correlations between individual BraincheX and CERAD-Plus subtests were variable, however, did not always align with the expected cognitive domains. This may reflect overlap between cognitive domains, differences in task characteristics between the digital and traditional assessments, and the limited sample size of this pilot study. Further research in larger samples is needed to establish the domain-specific validity of individual BraincheX subtests and the BraincheX total score.

BraincheX´s self-administration and partly automated scoring is designed to eliminate the need for an experienced examiner, reduce administrative time and enhance objectivity through automated scoring. In this current study, the BraincheX test battery was administered under controlled conditions with support from an experienced psychologist to ensure standardized testing procedures. Therefore, the current findings support the feasibility and validity of BraincheX in a supervised setting. While the self-administered design and automated scoring have potential to facilitate independent test administration, streamline clinical workflows, and improve scalability, these aspects warrant further investigation in unsupervised and real-world settings. The digital format may reduce rater bias, provide immediate results, improve reliability of test results across experimenters and settings. These features may position BraincheX as an efficient and scalable assessment tool for individuals with suspected early cognitive impairment.

The subtest correlation analysis supports the coverage of cognitive subdomains of BraincheX. Given the exploratory nature of the subtest analyses and the absence of correction for multiple comparisons, individual significant findings should be interpreted cautiously until replicated in larger independent samples. Moderate to strong associations between executive tasks (e.g., TMT-B, Clock Test) and memory-related CERAD subtests (e.g., Word List Learning, Recognition) suggest overlapping constructs. Language-related subtests, such as Animal Naming and Boston Naming, were significantly associated with both memory tasks. Similarly, correlations with visuospatial subtests (e.g., Figure Recall, Figure Drawing) further demonstrate that BraincheX covers multiple cognitive subdomains relevant to early AD pathology. These findings may underscore the battery’s construct validity and its ability to capture a multidimensional cognitive profile in a brief, digital format. Our findings align with previous research demonstrating that digital cognitive assessments can match conventional paper-based diagnostics in detecting MCI and dementia [22, 23]. In particular, BraincheX’s non-verbal and self-administered format intends to address language and cultural barriers, and to support inclusivity across diverse populations. Furthermore, the digital provision intends to facilitate access in underserved or remote areas, enabling earlier cognitive monitoring and intervention. BraincheX’s ability to deliver immediate feedback also positions it as a valuable tool for longitudinal cognitive tracking in clinical practice. Additionally, BraincheX may help to identify patients suitable for disease modifying treatments more efficiently. The expected implications of disease modifying treatments foresee, that there will be a significant change in need of diagnostic assessments [24]. BraincheX could contribute to streamlining both patient management and care pathways overall. As a result, modified treatment can become more accessible.

Despite these advantages, ethical concerns must be carefully managed, particularly regarding the automated delivery of potentially distressing results [25, 26]. Structured communication protocols and clinical follow-up by trained clinicians are essential to avoid anxiety and misinterpretation. Additionally, BraincheX’s integration into clinical workflows must be clearly defined, emphasizing its role as a tool for detecting signs of cognitive impairment—not as a standalone diagnostic instrument. Clinical diagnosis of AD should be based on a comprehensive assessment that includes BraincheX alongside other clinical evaluations, imaging, laboratory tests, and patient history.

Disparities in digital literacy remain a challenge [27, 28]. During the administration of BraincheX, the study team informally observed that some participants expressed hesitation or declined participation. Although these observations were not systematically documented, previous studies have identified low confidence with digital assessments, limited technological exposure, and age-related factors as potential barriers to digital cognitive assessments [27, 29, 30]. Future research will systematically assess usability, digital affinity, and subjective workload to identify barriers and optimize user-centered design.

### Limitations

This study represents the first stage in the comprehensive validation process of the BraincheX test battery. Several study specific limitations must be acknowledged. The current investigation included both amyloid negative cognitively healthy individuals and participants with early Alzheimer’s disease (AD). The inclusion of a mixed cohort was intentional, as it enables the examination of the test battery’s discriminative capacity between cognitively unimpaired and clinically impaired individuals from the outset. This approach provides preliminary insight into diagnostic sensitivity and supports the development of a test that is valid across a broad cognitive spectrum rather than only in healthy populations. Construct validity can be supported by the fact that the individual subtests assess distinct cognitive domains, analogous to established neuropsychological instruments such as the CERAD-Plus or RBANS. However, test–retest reliability and further validation across larger and more diverse samples remain necessary steps for future studies. Moreover, validating BraincheX across a broader range of cognitive disorders—such as Parkinson’s disease, stroke, and depression—could substantially enhance its clinical applicability and utility.

Second, the sample size of amyloid negative cognitively healthy participants was limited (*n* = 29), restricting the ability to calculate reliable normative data and to assess the effects of demographic variables such as sex, age, and education. The observation that only education showed a significant influence on overall performance, while age affected only one subtest and sex had no measurable effect, may be attributable to the small and non-representative normative sample rather than to independence from these factors. The regression-based normative model was derived from a relatively small healthy control sample and should therefore be considered preliminary. The computation logic underlying the BraincheX total score will be re-evaluated in the next development phase to further enhance its robustness and interpretability. The scoring procedure will rely on standardized *z*-scores obtained through a Huber-transformation framework and subsequently scaled using the Φ(*z*/τ) function. This approach is expected to yield an approximately normal distribution while reducing the influence of outliers, thereby improving comparability across participants. Once a larger normative dataset becomes available, the scoring algorithm will be re-evaluated and refined accordingly.

Third, while the BraincheX battery is designed for self-administration, testing in the current study took place under controlled conditions with a trained psychologist present. Although participants were encouraged to complete the battery as independently as possible, assistance was provided when questions arose. Thus, the results may not fully reflect real-world usability, and further studies are needed to evaluate autonomous administration in home and clinical settings.

Fourth, the time interval between CERAD-Plus and BraincheX assessment ranged from 0 to 12 months (M = 4.9). During this period, changes in cognitive performance may have occurred, particularly in individuals with MCI or early AD. Consequently, the observed associations between BraincheX and CERAD-Plus may have been influenced by disease progression or other temporal factors. Although previous studies have suggested that similar assessment intervals have only a limited impact on cognitive test performance, the non-concurrent administration of the two instruments should be considered when interpreting the reported convergent validity [31].

Another limitation is that the statistical analyses were not independently verified by an external investigator. Furthermore, blinding of the assessors to the clinical diagnosis was not feasible within the present study design.

Finally, since the tool has not yet been validated on a large sample of cognitively healthy participants, the current findings in individuals with early AD should be interpreted with caution. As can be seen in Fig. 1 individual outliers may have influenced the distribution of some subtest scores and, consequently, the derived normative data. A thorough validation, including detailed analysis of construct and criterion validity, test–retest reliability, and establishment of normative data, is required before application in clinical practice. Given its intended self-administered format, future research should specifically examine the feasibility and reliability of independent completion among individuals with mild cognitive impairment (MCI) and dementia to ensure usability across different cognitive levels.

## Conclusion

This study demonstrates BraincheX as an efficient and accessible digital tool for detecting cognitive impairment, particularly in Alzheimer’s disease. Its good internal consistency and strong convergent validity with the CERAD-Plus total score may support its role as a digital alternative to traditional assessments. Automated administration and scoring may enhance clinical consistency and scalability, while the non-verbal, culturally neutral format increases accessibility. Findings should be interpreted in the context of the pilot study design and the relatively small sample size. Further validation in larger cohorts is required to evaluate BraincheX’s potential role in differentiating individuals with early Alzheimer’s disease from healthy controls.

## Supplementary Information

Below is the link to the electronic supplementary material.

Supplementary Material 1

## Data Availability

The datasets generated and/or analyzed during the current study are available from the corresponding author on reasonable request.
